# Risk factors of malaria transmission in mining workers in Muara Enim, South Sumatra, Indonesia

**DOI:** 10.1038/s41598-023-40418-9

**Published:** 2023-09-07

**Authors:** Hamzah Hasyim, Wita Citra Dewi, Risva Aprina Fitri Lestari, Rostika Flora, Novrikasari Novrikasari, Iche Andriyani Liberty, Heni Marini, Ahmed Elagali, Siti Herlinda, Fadhilah Eka Maharani

**Affiliations:** 1 Faculty of Public Health, Universitas Sriwijaya, Palembang, Indonesia; 2 Faculty of Medicine, Institute for Occupational Medicine, Social Medicine and Environmental Medicine, Goethe University, Frankfurt Am Main, Germany; 3 Faculty of Medicine, Department of Public Health and Community Medicine, Universitas Sriwijaya, Palembang, Indonesia; 4 School of Biological Sciences, The University of Western Australia, Perth, Australia; 5 Minderoo Foundation, Perth, Australia; 6 Faculty of Agriculture, Department of Plant Protection, Universitas Sriwijaya, Palembang, Indonesia; 7 Research Center for Sub-Optimal Lands (PUR-PLSO), Universitas Sriwijaya, Palembang, Indonesia; 8 Faculty of Mathematics and Natural Sciences, Biology Department, Universitas Sriwijaya, Palembang, Indonesia

**Keywords:** Ecology, Health care, Health occupations

## Abstract

Eliminating malaria by 2030 is stated as goal three in the UN’s Sustainable Development Goals (SDGs). However, malaria still remains a significant public health problem. This study aims to identify the factors determining malaria transmission in artisanal or small-scale miner (ASM) communities in three villages: Tanjung Agung, Tanjung Lalang, and Penyandingan, located in the Tanjung Enim District, Muara Enim, South Sumatra, Indonesia. Researchers conducted a cross-sectional study involving 92 participants from the study area. They used a logistic regression model to investigate the risk factors related to malaria occurrence. The multivariable analysis revealed that age (Adjusted Prevalence Ratio (APR) = 7.989 with 95% CI 1.724–37.002) and mosquito breeding (APR = 7.685 with 95% CI 1.502–39.309) were risk factors for malaria. On the other hand, higher education (APR = 0.104 with 95% CI 0.027–0.403), the use of mosquito repellent (APR = 0.138 with 95% CI 0.035–0.549), and the condition of house walls (APR = 0.145 with 95% CI 0.0414–0.511) were identified as protective factors. The current study highlights age and mosquito breeding sites as risk factors for malaria. Additionally, higher education, insect repellent use, and the condition of house walls are protective factors against malaria. Therefore, reducing risk factors and increasing protective measures through effective communication, information, and education are highly recommended to eliminate malaria in mining areas.

## Introduction

Malaria infects around 200 million people and causes 400,000 deaths annually in 90 countries. The World Health Organization (WHO) has targeted eliminating malaria in 35 countries by 2030^1^. Eliminating malaria by 2030 is stated in the third goal of the Sustainable Development Goals (SDGs). However, malaria is a significant public health problem in Indonesia^2–5^.

The main requirements for WHO certification include an Annual Parasite Incidence (API) of < 1 per 1000 population, a Positivity Rate (PR) of < 5% (positive malaria/blood preparations examined), and zero incidences of indigenous cases (malaria transmitted by local *Anopheles* mosquitoes carrying *plasmodium*) for three consecutive years^6^. Malaria elimination certification necessitates interrupting local transmission for all human malaria parasites^7^.

From 2009 to 2022, Muara Enim Regency witnessed a decline in the API. The API figures API’s were 0.91 in 2009, 0.79 in 2010, 0.54 in 2011, 0.62 in 2012, 0.47 in 2013, 0.22 in 2016, 0.1 in 2017, and 0.1 in 2018. Subsequently, API figures for the three most recent years were 0.01, 0.003, and 0.005 in 2020, 2021, and 2022 respectively^8^.

Eliminating malaria in Muara Enim, South Sumatra, is challenging due to the presence of many mosquito species, particularly some of the main malaria vectors, in aquatic habitats created by human activity, such as puddles and ditches^9^. In South Sumatra, Indonesia, *Anopheles barbirostris*, *An. tesselatus*, *An. subpictus*, *An. nigerrimus*, *An. kochi*, *An. umbrosus*, *An. barbumbrosus*, and *An. maculatus*, have been found, with *An. sinensis and An. vagus* confirmed as vectors in Muara Enim Regency and *An. nigerrimus*, *An. umbrosus*, *An. letifer*, and *An maculatus* in OKU Selatan Regency^10,11^. Additionally, artisanal coal mining plays a significant role in the mobility of miners, particularly those from malaria-endemic regions. These miners may carry the *plasmodium* parasite from their original regions (imported cases) or experience malaria relapses due to inadequate medication, posing a risk of malaria transmission at the mining site and among the surrounding population.

Studying malaria transmission in artisanal or small-scale miner (ASM) communities is crucial as they represent a high-risk group^12^. Small-scale mining areas are particularly susceptible to malaria transmission^13^. For example, miners in Aceh, Indonesia, are at risk of contracting malaria^14^. Other studies have also indicated that mining areas are more vulnerable to malaria^15^, with consistent evidence of malaria vectors thriving in mining areas, as seen in the highlands of West Kenya^16^. Malaria risk factors in mining areas include socio-economic and behavioural factors^17^, while changes in land use can impact the spread of malaria^18,19^.

On the other hand, although the ASM population is unique in malaria elimination, little is known about malaria in miners in Guyana^20^. So, malaria transmission in miners is interesting to investigate. Over five years, the Annual Parasite Incidence (API) at Tanjung Agung Health Center, Muara Enim, decreased in 2018 to 0.7, followed by 0.49 in 2019 and 0.05 in 2020. However, malaria is still spreading in the Tanjung Agung Sub-District, where most ASM areas still need to be studied. The API was taken from the malaria surveillance information system (SISMAL). Therefore, this study aims to bridge knowledge gaps by exploring the factors contributing to a higher likelihood of malaria in this region.

## Results

### Participant characteristics and demographics

Each of the following research variables was examined using univariate analysis: age, sex, length of service, years of service, level of education, the habit of using mosquito nets, the existence of mosquito breeding and resting places, knowledge, attitude, behaviour, and home condition factors. Furthermore, we summarise the basic factors that describe the socio-demographic characteristics of the study participants. This was carried out to examine the association between these variables and miners’ malaria, as shown in Table 1.

**Table 1:** Bivariate analyses of baseline socio-demographic characteristics of participants (n = 92).

Variable	Malaria				PR; 95% CI (lb-ub)	*p* value
	Positive		Negative			
	n	%	n	%		
Age						
< 35 years old	3	8.57	32	91.43	1.33 (0.95–1.85)	0.163
≥ 35 years old	11	19.30	46	80.70		
Gender						
Woman	4	13.33	26	86.67	1.07 (0.74–1.54)	
Man	10	16.13	52	83.87		0.724
Years of service						
< Five years	1	2.70	36	97.30	1.72 (1.34–2.21)	0.006
≥ Five years	13	23.64	42	76.36		
Length of working						
< Eight hours	2	5.00	38	95.00	1.67 (1.23–2.26)	0.017
≥ Eight hours	12	23.08	40	76.92		
Education						
High (senior high school)	8	25.00	24	75.00	0.61 (0.33–1.15)	0.054
Low (primary school and junior high school)	6	10.00	54	90.00		
Mosquito net						
Yes	11	22.45	38	77.55	0.41 (0.14–1.16)	0.033
Not	3	6.98	40	93.02		
Using mosquito repellent						
Yes	11	25.58	32	74.42	0.36 (0.13–1.00)	0.005
Not	3	6.12	46	93.88		
Out of the house						
Not	3	8.57	32	91.43	1.33 (0.95–1.85)	0.163
Yes	11	19.30	46	80.70		
Self-medication						
Yes	12	17.65	56	82.35	0.50 (0.13–1.91)	0.278
Not	2	8.33	22	91.67		
Knowledge						
High	12	22.64	41	77.36	0.30 (0.08–1.10)	0.028
Low	2	5.13	37	94.87		
Attitude						
Good	10	24.39	31	75.61	0.47 (0.20–1.10)	0.021
Not good	4	7.84	47	92.16		
Practice						
Good	7	17.95	32	82.05	0.84 (0.48–1.47)	0.535
Not good	7	13.21	46	86.79		
Mosquito breeding						
No risk	2	5.56	34	94.44	1.51 (1.13–2.02)	0.036
At risk	12	21.43	44	78.57		
Resting place						
No risk	1	3.13	31	96.88	1.54 (1.22–1.94)	0.014
At risk	13	21.67	47	78.33		
House wall condition						
Eligible	9	25.71	26	74.29	0.53 (0.26–1.10)	0.021
Not eligible	5	8.77	52	91.23		
The ceiling of the house						
Eligible	8	16.00	42	84	0.92 (0.48–1.77)	0.816
Not eligible	6	14.29	36	85.71		
House floor condition						
Eligible	8	25.00	24	75.00	0.61 (0.33–1.15)	0.054
Not eligible	6	10.00	54	90.00		

The 95% CI of percentage in bivariate analysis.

*lb* Lower 95% confidence boundary of cell percentage, *ub* Upper 95% confidence boundary of cell percentage.

In our study population, 78 (84.78%) respondents had no malaria or tested negative for malaria, whereas 14 (15.22%) were malaria positive. Among these, 38.04% were under the age of 35, while 61.96% were over the age of 35. The study included 62 men and 30 women. Respondents with less than five years of service accounted for 40.22%, while 59.78% had more than five years of service. Out of the fifty-two respondents, 56.52% worked less than eight hours, while 43.48% worked eight hours or more. Regarding education, 60 respondents were primary and junior high school graduates (65.22%), while 34 were senior high school graduates (34.78%). 53.26% of the respondents used mosquito nets, while 46.74% did not.

Additionally, 46.74% used mosquito coils, while 53.26% did not. 38.04% did not leave their houses, while 61.96% did. Among 68 respondents, 73.91% self-medicated, whereas 26.09% did not. Fifty-three respondents (57.61%) were knowledgeable, while 42.39% had insufficient knowledge. Furthermore, 44.57% had a good attitude, while 55.43% did not. 57.61% of the 92 respondents had poor practices, whereas 42.39% had good practices. The presence of breeding sites indicated that most miners were at risk (60.87%). 65.22% of respondents lived near at-risk resting sites. 61.96% of the respondents had ineligible house walls, while 38.04% had qualified ones. Moreover, 55.40% of the respondents met the eligibility criteria for their dwelling condition, while 44.60% did not. Finally, 65.22% of the respondents had non-eligible house walls, while 34.78% had eligible ones.

Based on the bivariate analysis table, several variables showed statistical significance with the prevalence of malaria. There is a statistically significant relationship between years of service (*p* = 0.006) and the length of time working with malaria status (*p* = 0.017). Furthermore, the chi-square test demonstrated a statistically significant relationship between mosquito net usage (*p* = 0.033) and the use of mosquito repellent (*p* = 0.005) with malaria. Moreover, the chi-square test revealed a statistically significant relationship between knowledge (*p* = 0.028) and attitude (*p* = 0.021) towards malaria. Additionally, mosquito breeding (*p* = 0.036) and resting place (*p* = 0.014) were found to be related to malaria. Finally, this study indicated that house wall condition (*p* = 0.021) and malaria showed statistical significance.

We selected the backward stepwise method for multivariable regression models, starting with a saturated model and gradually reducing variables to find the most appropriate model that explains the data. Variables were excluded during the backward stepwise process when their *p* values became insignificant. In the bivariate selection, a *p* value < 0.25 was used, and during the modelling phase, the variable with the highest *p* value was excluded until a model with a *p* value < 0.05 was obtained. Since these variables may influence one another, the variable with the most significance is considered the dominant variable on the outcome. Thus, the prevalence ratio is multivariable and referred to as adjusted. Furthermore, a multivariable analysis was performed to identify the dominant risk factors for malaria, as shown in Table 2.

**Table 2:** Factors associated with malaria prevalence in the low endemic area (n = 92).

Research variables	PR crude (95% CI)^a^	*P* value	PR adjusted (95% CI)^b^	*P* value
Age				
< 35 years old				
≥ 35 years old	1.33 (0.95–1.85)	0.163	7.98 (1.72–37.00)	0.008
Education				
High (senior high school)				
Low (primary school and junior high school)	0.61 (0.33–1.15)	0.054	0.10 (0.02–0.40)	0.001
Using mosquito repellent				
Yes				
Not	0.36 (0.13–1.00)	0.005	0.13 (0.03–0.54)	0.005
Mosquito breeding				
No risk				
At risk	1.51 (1.13–2.02)	0.036	7.68 (1.50–39.30)	0.014
House wall condition				
Eligible				
Not eligible	0.53 (0.26–1.10)	0.021	0.14 (0.04–0.51)	0.003

*Ref.* The reference category is represented in the contrast matrix as a row of one.

^a^Crude prevalence ratio (PR).

^b^Adjusted prevalence ratio (APR).

A multivariable analysis was carried out to determine the most influential factors. Finally, multivariable analysis showed that age was the main risk determinant factor, with a *p* value of 0.008. Higher education, insect repellent, and the house’s walls were protective factors, with PR < 1.

### Principal findings

It is crucial to study malaria transmission in smallholder mining areas, as these areas are at high risk for malaria transmission. While many risk factors increase the possibility of malaria transmission in traditional mining, there was relatively little evidence in this study regarding malaria in miners, who are one of the populations targeted for malaria elimination. The major finding of this current research revealed that smallholder mining areas were risky areas for malaria transmission. Out of the 14 patients infected, 12 had *plasmodium falciparum*, and the remaining two had a *plasmodium mix. *(*P. falciparum* + *P. vivax*). Based on a Rapid Diagnostic Test (RDT) and microscopy results, *P. falciparum* was the most frequent parasite in the study area.

The current study identified the age of small-scale mining respondents and mosquito breeding sites as risk factors for malaria. Moreover, higher education, insect repellent use, and the condition of the house’s walls were found to be protective factors against malaria in the study area. In the multivariate analysis, participants over 35 years of age were 7.98 times more likely to have malaria than those under 35 years of age (adjusted PR: 7.98; 95% CI 1.72–37.00; *p* value: 0.008) after adjusting for education, mosquito repellent use, mosquito breeding, and the condition of the house walls. Additionally, the use of insect repellent was closely related to a reduced risk of malaria (adjusted PR: 0.13; 95% CI 0.03–0.54; *p *value 0.005).

Community mining activities, or ASM, have provided benefits because they are considered one of the poverty alleviation strategies^21^. ASM has played an essential role in the community’s economy and improved people’s livelihoods in various studies^22–25^. However, malaria transmission has been observed in ASM areas, posing health risks and environmental burdens due to land degradation^26^. Malaria transmission in the ASM area can increase malaria prevalence^27^. The spike in *P. falciparum* malaria observed in Guyana was most likely driven mainly by increased ASM activity^28^. The abundance of mosquito species was higher in ASM areas, leading to increased malaria prevalence^29^. Environmental degradation caused by mining and climate change has also led to the re-emergence of malaria in transmission hotspots^30^. Studies have shown a link between age and malaria transmission, where malaria is more likely as people get older^31–33^.

Regarding the breeding site issue, mining activities have produced artificial breeding grounds for vector mosquitoes^34^. For instance, Brazilian gold miners on the Amazon border dug pits and ditches, which were then abandoned, resulting in stagnant water that provided ideal habitat for mosquito breeding and malaria transmission^35^. Reducing breeding sites can decrease mosquito larval habitats and adult production^36,37^.

In this study, respondents lived either in the vicinity of their workplace or nearby, with some residing at their workplace for extended periods by arranging basic accommodations within the work area. Notably, certain miners lived in the forest. *An. sinensis and An. vagus* were confirmed as vectors in Muara Enim Regency, with sporozoites identified in their samples. Both species were active between 9 p.m. and 4 a.m. and preferred different biting locations. Mosquito-borne infections are more likely to occur at the workplace, especially for miners working without personal protective equipment, such as long-sleeved clothing or mosquito-repellent lotion.

Water-stored *Anopheles* mosquito eggs hatch into larvae and adult mosquitoes. Female mosquitoes need blood to feed their eggs. They search for “food” from dusk to dawn and can spread the *plasmodium* parasite. Mosquito numbers peak during and after the rainy season. Densely inhabited places increase malaria outbreaks. Malaria is more widespread at work than home since some illegal employees in Muara Enim, a mining area, work at night without protection. Working hours are from morning to evening, but some employees work until late at night. Additionally, some miners live or stay in the forest.

Regarding the socio-demography issue, another study in south-eastern Nigeria showed that higher education increases malaria protection knowledge and practices^38^. Other research shows that the spread of malaria can be reduced or eliminated using spatial repellents (SR)^39^. In this study, the condition of the house walls was identified as a protective factor against malaria in the study area, which aligns with the results of another study that examined housing and malaria^40^. In Uganda, good housing construction reduced malaria risk by limiting mosquito vectors’ entry^41^.

### Explanatory variables

The current study showed that the infected study participants, who work in smallholder mining, were aged between 18 and 65 years, an average of 43 years, and exposed to malaria, with the majority (61.96%) aged 35 years. So this group needs to be a priority for preventing malaria. Furthermore, the current study showed that mosquito breeding sites indicated that most mining workers were at risk (60.87%), and so did the availability of mosquito resting areas (65.22%) associated with breeding sites. ASM activities resulted in significant environmental changes, including the creation of mosquito breeding sites, which allow disease vectors to multiply—associated with malaria transmission^42^. In another study in Moneragala, mining pits can increase the proliferation of malaria vectors^43^.

Similarly, mosquito breeding sites near homes are common in sub-Saharan Africa and cause malaria infection^44^. Another study found that land cover change is a significant cause of rising highland temperatures in Africa and the colonisation of malaria vectors. It also increases the sporogony development rate, survival of adult vectors, and risk of malaria in the highlands^45^. Efforts to reduce standing water and eliminate mosquito breeding sites can drastically reduce the *Anopheles* mosquito population^46^. Thus, it was recommended to eliminate mosquito breeding places in the mining area^47^. This current research demonstrates knowledge related to malaria transmission in ASM. In this respect, it is in line with several studies which show that public health education is essential for malaria control^48,49^. Previous studies showed that public health education is crucial for malaria control. This study showed that respondents’ overall knowledge score, attitude, and practice level towards malaria were relatively good. Thus, health education to raise the community’s awareness about the disease must address the gaps identified by this study^50^. Recognising social and behavioural risk factors and knowledge gaps in malaria-endemic regions is crucial to formulating practical disease prevention guidelines. Health professionals in malaria-endemic areas should be taught to provide better counselling to address cultural behaviours such as nighttime outdoor time, inappropriate bed net use, and irregular pesticide use during sleep to overcome local malaria knowledge gaps^51^. Effective disease-fighting tactics depend on population knowledge and behaviour. However, public authorities should continue to raise awareness about malaria prevention and treatment because more than half of the population self-medicates, which could perpetuate the disease and drug resistance^52^. Workers risk becoming infected if they do not use personal protective equipment, such as long-sleeved clothing or mosquito-repellent lotion while working in the forest. It is crucial to enhance respondents’ knowledge, attitudes, and practices (KAP) to prevent malaria transmission; National Malaria Control Programs should promote access to education and assist communities in adopting better malaria prevention and control practices.

In Yunnan Province, China, low education and risky behaviour lead to a high malaria incidence^53^. This study on ASM showed that respondents have a low level of education, and there is a need to increase specific knowledge about preventing malaria transmission. Improving accurate public knowledge and prompt treatment-seeking behaviour for malaria elimination for early diagnosis and treatment are essential^54^. Lack of access to and adherence to artemisinin-based combination therapy (ACT) is an obstacle to the development of successful malaria treatment, which is also influenced by patient knowledge, attitudes, and beliefs^55^.

In addition, several studies have shown that using malaria repellent prevents malaria transmission^56,57^. Other studies highlight the need to strengthen *plasmodium* infection prevention and control strategies using personal mosquito repellents^58^. Furthermore, mosquito coils are popular in malaria-endemic countries^59^.

The current study showed that most miners had a poor level of education (65.22%), did not use mosquito coils (53.26%), and had a habit of leaving the house at night (61.96%), all of which are risk factors for malaria. It is in line with another study which revealed that outdoor miners were susceptible to mosquito bites and that modifying the house reduces malaria effectively^60^.

Home screening can reduce mosquito density^61^. Malaria is disappearing from areas that have been endemic for centuries, such as England’s south coast, because of better housing^62^. In Baringo District, the malaria vector prefers open roofs. Homes with thatched roofs have more malaria vectors than metal roofs^63^. So, future research should evaluate the protective effect of adding certain home features^64^. The current study showed that the state of the house’s walls (61.96%) and the ceiling (55.40%) were related to malaria transmission. It is similar to another study that showed a 47% lower malaria infection rate in improved (modern) homes compared to traditional ones^64^. In contrast, poor building construction and individual behaviour in Ghana are reasons for the high prevalence of malaria^65^.

## Method

### Study design and setting

The cross-sectional survey was conducted using a structured questionnaire from May to July 2022. All methods followed relevant guidelines or regulations^66^, including compliance with ethical requirements and specifically sampling without biological specimens. The interviews were conducted by obtaining informed consent; the respondents signed the consent form after understanding the aims and objectives of the research and voluntarily providing data to the researcher without coercion, and the identity of the respondents was made anonymous by the researcher to maintain confidentiality.

The study was approved by the Health Research Ethics Committee of the Faculty of Public Health, Universitas Sriwijaya, with ethical approval No: 313/UN9.FKM/TU.KKE/2022. Participation in this study was voluntary in the field. All analyses were performed using participant identification codes to ensure maximum confidentiality.

**Figure 1:**
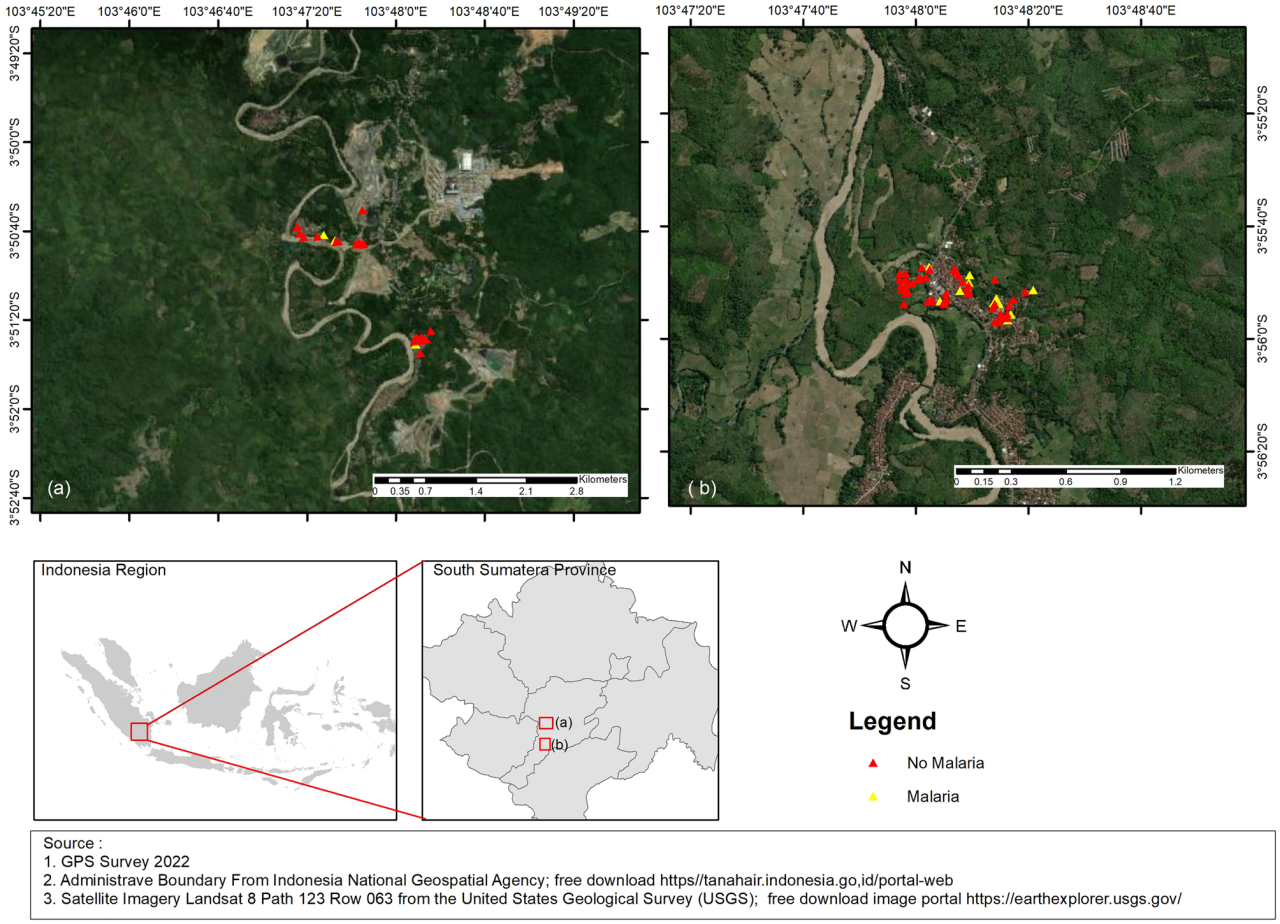
Study area.

Using a purposive method, the investigator chose three villages, namely Tanjung Lalang, Tanjung Agung, and Penyandingan, in Tanjung Agung Sub-District, Muara Enim District. The study areas were selected based on information about the sites with the highest mining activities. The study areas are shown in Fig. 1

### Sample size

This study uses a hypothesis test for two population proportions (two-sided test) with a level of significance of 95%, a type I error (α) of 5%, and a test power of 80%^67^.

It utilised P1 and P2 from previous studies for sample size determination and selected variables. Based on the literature review, the anticipated population proportion 1 (P1) for patients who do not use mosquito nets and have malaria is 0.79, while the anticipated population proportion 2 (P2) for patients who use mosquito nets and have malaria is 0.50. Therefore, the minimum sample size for each group in this study is 42 people. The results are multiplied by two because a two-proportion sample formula is used, resulting in an estimated total minimum sample size of 84 people. To account for potential dropouts and other factors, the sample size was increased by 10% to 92.4 and rounded to 92 respondents. Thus, the total sample calculation yielded 92 people. This recent study used a cross-sectional study design and revealed that 78 (84.78%) respondents had no malaria, whereas 14 (15.22%) had malaria.

### Sampling technique

**Figure 2:**
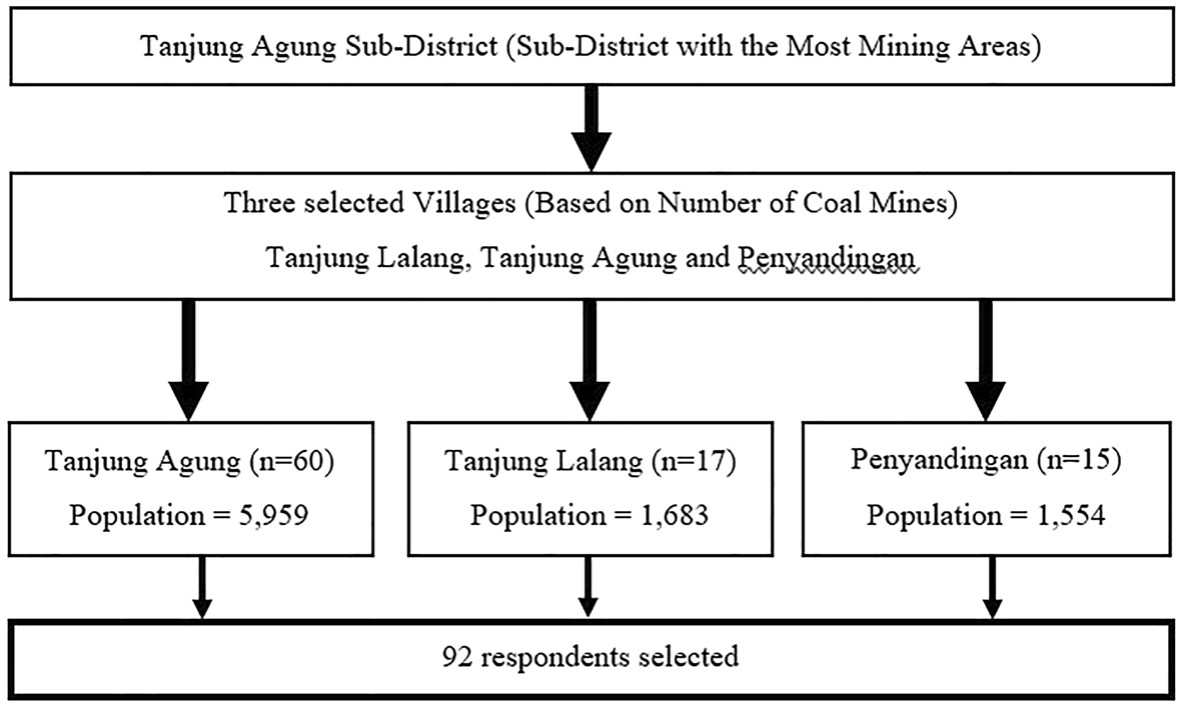
Flowchart of a sampling frame of the study.

This study employed a purposive sampling technique to select clusters or villages. The village with the highest number of miners was chosen as the criteria for selection. The sampling method was done randomly in each village using the existing sampling frame in each chosen village. The number of samples taken in each village was determined proportionally, where the minimum sample obtained represents the population of artisanal small-scale mining (ASM) in these three villages. Due to social and environmental factors affecting malaria in small-scale mining areas, there were malaria-positive cases in this population, making the respondents at risk for malaria transmission. An illustration of the sampling technique is shown in Fig. 2

Using Muara Enim District’s Central Bureau of Statistics data, the investigator made a cluster fraction sample and grouped people in each village. Finally, the respondents were chosen using two-stage cluster sampling in the study area. The current study has improved Internal and external validity. The population’s livelihoods in the villages are heterogeneous; apart from labourers and employees, they also contribute to plantations, livestock, fisheries, farmers, and ASM. More mining locations exist in these three selected villages than in other areas.

### Data collection

After random sampling, inclusion/exclusion criteria are based on the at-risk population. Thus, the eligibility criteria or inclusion criteria of this current study were that the respondent has lived in Muara Enim Regency for more than six months, is an illegal miner, can communicate properly and is ready to be a respondent. While the exclusion criteria are if the respondent does not finish the questionnaire and the sample respondents cannot speak effectively.

Epidemiological, socio-demographic characteristics, behavioural risk factors and observational environmental data were collected using a structured questionnaire. This questionnaire’s validity and reliability were tested on 30 respondents who live in Darmo village, which has mining activities. The respondents have the same characteristics as the sample study. After getting a valid and reliable questionnaire, the questionnaire was used for the respondents to collect information on risk factors of malaria occurrence in ASM in the study area. Eligible participants were informed about the general objectives. Oral consent was obtained before the interview, and information was recorded on paper case record forms. Investigators interviewed 92 miners who met the requirements to participate in the study. The interview, a structured questionnaire with open-ended and closed items, was conducted face-to-face.

### Scope of variables

The data was processed using Stata software: on the dependent variable (malaria occurrence), respondents who did not experience malaria were coded 1, and respondents who had or were experiencing malaria were given code 2 in this study, conducted in 2022. Malaria infection (positive malaria) are patients with symptoms of malaria who have been declared positive based on rapid diagnostic tests (RDTs) test and microscopy. In this study, the respondents who did not experience positive malaria were those who had negative malaria and were also healthy. In the questionnaire, for the dependent variable, the investigator asked respondents, “Had the respondents ever had blood drawn for malaria examination by a health worker?”. The answer was binary “Yes or Not”. If the answer was “Yes”, the next question was: had the respondents tested positive for malaria after examination by a health worker?

Likewise, the independent variable, code “small,” is given to describe the variable in the “bad/low” condition or the “at-risk” group. This category is grouped under code 1. At the same time, the code “large” is given to describe variables in “good/high” conditions or groups with the category “no risk” with code 2.

### Socio-demographic characteristics of participants

All independent variables were coded as one risk category, with code 2 indicating no risk. The respondent’s age is recorded in years 35 (code 1) and 35 (code 2), then the years of service are divided using years five years and < 5 years and the division of working hours for respondents is calculated hourly 8 h and < 8 h in a day. The gender differences were divided into male and female and taken from the questionnaire. In this study, education is the highest level of education achieved by participants. Participants were considered highly educated after graduating high school and coded = 2. Participants who did not complete high school were classified as uneducated and assigned the code = 1.

### Behavioural risk factors

From the questionnaire, the use of mosquito nets is categorised as follows: the respondent who does not use mosquito nets at night when sleeping is given a code of 1. If the respondent uses a mosquito net, it is coded as 2. The habit of using mosquito repellent is then divided; if not or only occasionally using mosquito repellent, code 1 was assigned, and code 2 was assigned to respondents who used mosquito repellent. The habit of going out at night and taking self-medication uses code 1 if yes, or sometimes code 2 if you never go out at night and take self-medication. The respondents’ knowledge is then divided into lack of knowledge and high knowledge and code 1 and code 2 for those with high knowledge. Meanwhile, attitude and practice are categorised with code 1 if they are not good and code 2 if they are good.

### Environmental risk factors

Environmental factors are divided into two categories: outside and inside the home. In the outdoor environment, whether there is a breeding place for mosquitoes and a resting place for mosquitoes. The presence of a breeding and resting place is coded 1 if it is at risk or if the distance is 100 m from the respondent’s home location, and code 2 is not at risk if it is > 100 m. Regarding environmental elements, the condition of the house’s walls and the existence of the ceiling are significant. Additionally, the state of the house’s flooring Researchers assigned code 1 for a housing condition that does not match the requirements and is not an eligible house and code 2 for a housing condition that meets the requirements.

### Factors associated with malaria occurrence using a structured questionnaire.

Information was primarily collected from respondents who were mining workers. The dependent data from a structured questionnaire included epidemiological data on malaria occurrence; At the same time, the independent variable included respondents’ socio-demographic characteristics, including age, sex, years of service, length of work, and education level. In addition, behavioural risk factors included using a mosquito net, mosquito repellent, being out-of-the-house at night, self-medication, and KAP (knowledge, attitude and practice) also investigated. Furthermore, environmental risk factors included mosquito breeding, resting place, house wall condition, the existence of the house ceiling, and house floor condition. Another study found that bamboo/wood house walls, no insecticide, and a distance < 100 m from the mosquito breeding site were malaria risk factors^68^. The previous study showed environmental housing variables affect transmission. Mosquito breeding places near dwellings influence malaria transmission^69^. Household and neighbourhood-focused malaria interventions may assist high-burden nations. Targeted vector control methods like indoor residual pesticide spraying can help reduce malaria, address age-related differences in malaria outcomes, and ITN use^70^.

*Anopheles* larvae breed in former mining excavations, lagoons, swamps, buffalo puddles, ponds, ditches, rice and fields as breeding sites. A questionnaire and GPS measurements were used to determine whether each dwelling had a breeding site. Previously, *Anopheles* data were collected by the South Sumatra Provincial Health Office, Baturaja Litbangkes, and Muara Enim District Health Office.

### Data analysis

Data were analysed using descriptive statistics, chi-square and logistic regression analysis using STATA statistical software. Other data extracted were the availability of association between these variables and the occurrence of malaria in miners, provided in Tables 1 and 2, respectively.

### Descriptive analysis

Individual characteristics of respondents include age, sex, years of service, length of work, and education level. Furthermore, behavioural risk factors include a mosquito net, mosquito repellent, out-of-the-house at night, and self-medication, then KAP (knowledge, attitude and practice) are the goals of descriptive analysis.

### Bivariate analysis

The significance of the statistical relationship between each independent and dependent variable was analysed using the Chi-Square test by comparing the probability value (*p* value) with the alpha (α) = 0.05. If the *p *value is < 0.05, Ho (the null hypothesis) is rejected. It means there is a significant statistical relationship between the independent and dependent variables; if the *p* value > (0.05), Ho is accepted or failed to be rejected, meaning there is no significant statistical relationship between the independent and dependent variables.

### Multivariable analysis

The malaria risk severity was evaluated using crude and adjusted prevalence ratio (aPR)†. If the prevalence ratio (PR) is more than one, the probability of developing malaria increases.

### Limitations of research

In the study area, malaria transmission is influenced by various risk factors, although not all of them have been considered as research variables. This aspect presents an opportunity for further investigation. However, the current variables under study primarily pertain to social and environmental factors that impact malaria in small-scale mining areas. It is important to note that the study’s sample size is relatively small, which may introduce some bias in the results, particularly in assessing the proportion of malaria-positive cases. The sample size was determined using the sample formula for testing the two-proportion hypothesis, using data from previous studies regarding P1 and P2, as well as selected variables. This approach has improved the internal and external validity of the study, making it representative of artisanal mining in the three villages. In these villages, the residents’ livelihoods are diverse, involving activities such as smallholder plantations, livestock keeping, fisheries, farming, and mining. Interestingly, the study did not specify the respondents’ working hours, which could be associated with the risk of Anopheles mosquito bites. Despite this, it was found that respondents worked varying hours, with some working less than 8 h and others working 8 h or more. The majority of respondents (56.5%) opted for longer working hours to increase their daily income, as more hours of work resulted in higher wages. Hence, economic considerations drove many respondents to extend their working hours.The working hours typically span from morning until evening, although some individuals work until late evening. It is worth noting that An. sinensis tends to bite outdoors, while An. vagus prefers indoor biting. Both species are active from 9 p.m. to 4 a.m. These findings may have broader implications and can potentially be applied to other small-scale mining areas.

In summary, while additional risk factors may affect malaria transmission in the study area, the current research is primarily centered on the social and environmental factors impacting malaria in small-scale mining regions. Although small, the study’s sample size was carefully determined, enhancing the validity of the findings for artisanal mining in the three villages. Working hours play a role in malaria risk, with economic factors driving some respondents to work longer. Different mosquito species’ time preferences and biting behaviours shed light on the potential applicability of the study’s concept to other similar mining locations.

### Informed consent

I undersign a certificate that I have the written consent of the identifiable person or their legal guardian to present the cases in this scientific paper.

## Conclusions

Multivariable analysis revealed age and mosquito breeding as risk factors for malaria. A high level of education, the use of insect repellent, and the condition of the house’s walls are protective factors. Eliminating breeding sites in mining areas or avoiding direct contact between artisanal miners and vectors around the breeding sites can be facilitated by increasing knowledge, using insect repellent or protective clothing, and improving housing conditions as a protective factor. As an essential step towards eliminating malaria, generally, and at least in the research area, it is crucial to take preventive and promotional actions.

## Data Availability

The original contributions presented in the study are included in the article/supplementary material. Further inquiries can be directed to the corresponding author.

## Abbreviations

ACT: Artemisinin-based combination therapy
An.: *Anopheles*
API: Annual parasite incident
APR: Adjusted prevalence ratio
ASM: An artisanal miner or small-scale miner
KAP: Knowledge, attitude, and practice
SISMAL: Malaria surveillance information system
*p* value: The probability value
PR: Prevalence ratio
RDT: Rapid diagnostic test
*SDG*: Sustainable development goals
SR: Spatial repellent
WHO: World Health Organisation
